# Development of porcine rotavirus vp6 protein based ELISA for differentiation of this virus and other viruses

**DOI:** 10.1186/1743-422X-10-91

**Published:** 2013-03-22

**Authors:** Jiayi Zhu, Qing Yang, Liyan Cao, Xiujing Dou, Jianguo Zhao, Weijuan Zhu, Fan Ding, Ri-e Bu, Siqingaowa Suo, Yudong Ren, Guangxing Li, Xiaofeng Ren

**Affiliations:** 1 College of Veterinary Medicine, Northeast Agricultural University, 59 Mucai Street, Harbin, Xiangfang District, 150030, China; 2 School of Life Science, Inner Mongolia University for the Nationalities, Tongliao, Inner Mongolia, China; 3 College of Veterinary Medicine, Hunan Agricultural University, 1 Nongda Road, Changsha, Furong Disctrict, 410128, China; 4 State Key Laboratory of Reproductive Biology, Institute of Zoology, Chinese Academy of Sciences, Beijing, 100101, China; 5 Department of Health, Chinese Armed Police forest Force Command, Beijing, 100097, China

**Keywords:** PRV, VP6 gene, Cloning, Expression

## Abstract

**Background:**

The context and purpose of the study included 1) bacterial expression of viral protein 6 (VP6) of porcine rotavirus (PRV) and generation of rabbit polyclonal antiserum to the VP6 protein; 3) establishment of a discrimination ELISA to distinguish PRV from a panel of other porcine viruses.

**Results:**

The VP6 gene of PRV isolate DN30209 amplified by reverse transcription-PCR was 1356 bp containing a complete open reading frame (ORF) encoding 397 amino acids. Sequence comparison and phylogenetic analysis indicated that PRV DN30209 may belong to group A of rotavirus. Bacterially expressed VP6 was expressed in *E.coli* and anti-VP6 antibody was capable of distinguishing PRV from Porcine transmissible gastroenteritis virus, Porcine epidemic diarrhea virus, Porcine circovirus type II, Porcine reproductive and respiratory syndrome virus, Porcine pseudorabies virus and Porcine parvovirus.

**Conclusions:**

PRV VP6 expressed in E. coli can be used to generate antibodies in rabbit; anti-VP6 serum antibody can be used as good diagnostic reagents for detection of PRV.

## Background

Rotavirus, a member of family *Reoviridae*, is one of the causative agents of viral diarrhea in young children and animals worldwide [1-3]. Rotavirus is approximately 75 nm in diameter and has multilayered icosahedral protein capsid composed of an outer layer, an inner layer, and a core [4]. The virus genome is comprised of 11 segments of double-stranded RNA, encoding six structural proteins (VP1-VP4, VP6, VP7) and six nonstructural proteins (NSP) [5,6]. The outer capsid layer proteins of rotavirus, VP4 and VP7, may elicit neutralizing antibodies independently; in addition, the two proteins can be used to classify rotaviruses into P and G genotypes, respectively [7-9].

VP1, VP2 and VP3 proteins consist of the rotavirus core particles. VP1 was partially accessible to iodination in single-shelled particles [10]. The accession degree of VP1 to the immune system may affect the reaction of anti-PRV to VP1 protein [4]. VP2 is the most abundant structural protein in core particles [11]. Anti-VP2 serum is a good indicator of prior infection due to its high immunogenicity [12]. VP3 is a minor structural protein that may comigrate with the outer capsid protein VP4 in many gel systems and it may be involved in RNA replication; VP4 is cleaved to form VP5 and VP8 [4,11].

VP6 is the major structural protein in virus particles located on the outer surface of single-shelled particles, in addition, VP6 is known as a subgroup antigen [4]. VP6 is required not only for polymerase activity and but also for maintaining the proper structure of the viral core or the core protein-based transcriptional complex. Myristylation of VP6 is required for virus particle formation as well as targeting single-shelled particles to the ER membrane for budding [13]. As VP6 is both highly immunogenic and antigenic, it is the most frequently used in diagnostic assays to detect PRV [14]. Herein, we expressed the VP6 gene from a PRV Chinese isolate DN30209 by cloning this gene into a bacterial expression system. We then generated rabbit polyclonal anti-serum and ascertained its immunoreactivity to VP6 protein. Using the anti-VP6 antibody, we established a differentiating ELISA for PRV. These experiments provided basis for determination of PRV.

## Results and discussion

### Cloning and phylogeny of VP6 gene of PRV

**Figure 1:**
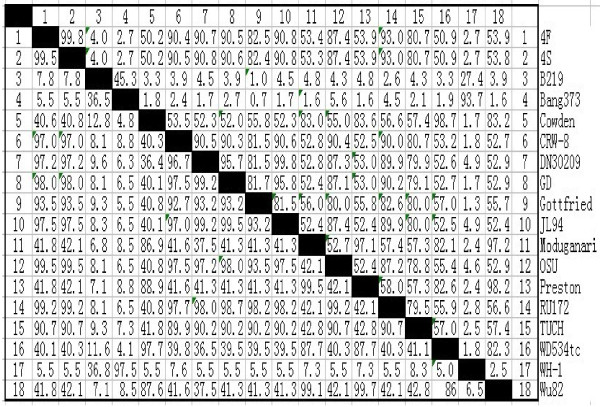
**Homologous identity comparison of PRV VP6 gene.** VP6 sequences of PRV isolates used in this study were subjected to multiple sequence alignments using the DNAStar software. Their grouping, name and origin are shown in Table 1.

**Figure 2:**
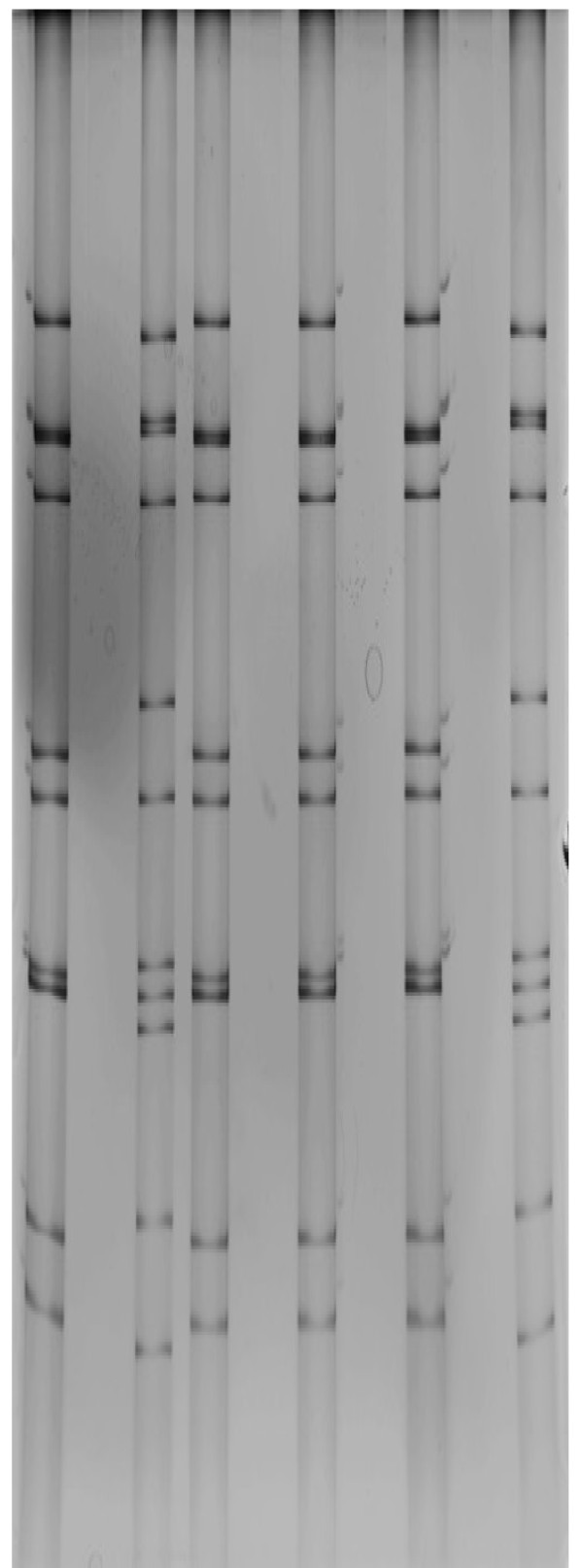
**RNA-Polyacrylamide gel electrophoresis (PAGE) of PRV.** RNA-PAGE profile shows long electropherotype migration pattern of PRV DN30209 (lane 1). Lane 2 is negative sample.

**Figure 3:**
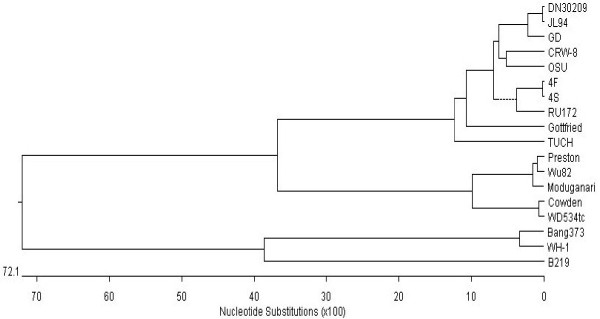
**VP6 amino acide-based phylogenetic tree.** A phylogenetic tree was constructed based on these PRV VP6 amino acid sequences using DNASTAR software.

PRV may cause piglet diarrhea andV P6 is an important structural component of viral particles. In this study, we cloned the VP6 gene from a Chinese PRV isolate DN30209. The sequencing results showed that the cloned VP6 gene was 1356 bp containing a complete open reading frame (ORF) encoding 397 amino acids. The sequence has been submitted to GenBank and was allocated a GenBank accession number (JN977137). Compared with PRV JL94, there were three mutations at N_94_H, G_126_R and I_182_V; compared with PRV GD, three mutations N_94_H, I_182_V and T_244_A were identified. As DN30209, JL94 and GD all were isolated from northern China, these viruses may be geographical prevalent in northern areas in China. Based on the randomly selected PRV reference strains, the VP6 amino acid sequences were compared. As shown in Figure 1, PRVs DN30209 and several PRV group A strains OSU, JL94 GD, CRW-8, 4F and 4S etc. had more than 90% homologous identity at amino acid level. RNA-PAGE analysis showed that the rotavirus represented long electropherotype migration pattern of porcine group A rotaviruses (Figure 2). Further phylogenetic tree analysis also indicated that PRV DN30203 located in the same clade with above-mentioned PRV group A strains (Figure 3). Therefore, we speculate that PRV DN30209 also belongs to group A of rotavirus. Nevertheless, it is necessary to do more serological and epidemiological investigations to get full understanding on the evolution of PRV in the future.

### Analysis of VP6 expression and immunoreactivities of its antibody

**Figure 4:**
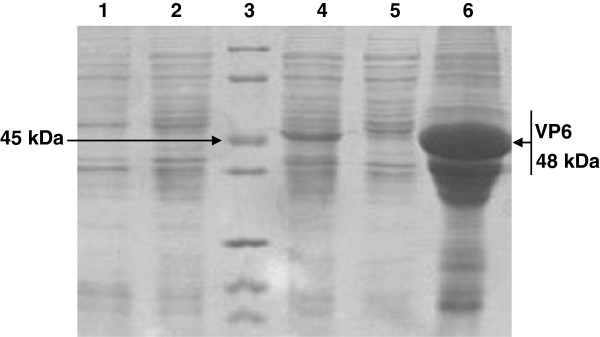
**Bacterial expression of VP6 protein in *E.coli.*** Expression of VP6 protein was detected by a Coomassie blue staining. PAGE gel screened with rabbit antiserum to the VP6 as the primary antibody. Lane 1, crude bacterial lysate from cells containing non-recombinant vector only; Lane 2, non-induced crude bacterial lysate from bacteria containing VP6 plasmid; Lane 3, protein marker; Lane 4 unlysed VP6-containing bacteria at 4 h post-induction; Lane 5, lysed VP6-containing bacterial supernatant at 4 h post-induction; Lane 6, lysed VP6-containing bacterial inclusion bodies. The size of protein marker and the VP6 protein is indicated.

**Figure 5:**
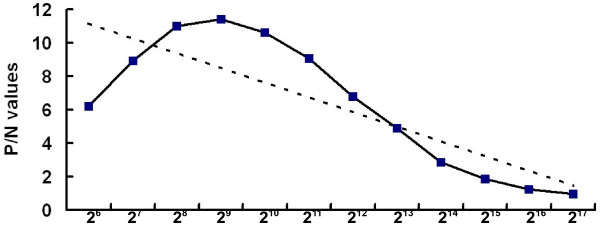
**Optimization of dilution of anti-VP6 antibody in ELISA.** Purified VP6 protein (5 μg/well) was diluted and coated in ELISA wells, and the serially diluted anti-VP6 antibody was used as primary antibody in subsequent ELISA. Dilutions of anti-VP6 antibody are indicated. The OD_490_ value of tested samples (P)/the OD_490_ value of negative control, coating buffer (N)>2 is judged as positive and shown as a curve; the broken-line is trend line.

**Figure 6:**
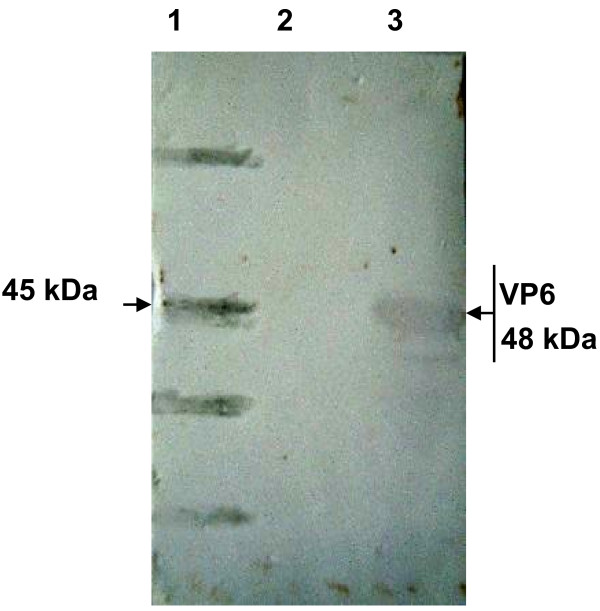
**Western blot of VP6 protein*.*** Empty vector-transforming bacterial protein (negative control) and VP6-bearing bacterial protein were transferred onto a nitrocellulose membrane, then, the membrane was consecutively incubated with the anti-VP6 antibody and HRP-conjugated secondary antibody. Their blot results are shown in lanes 2 and 3, respectively. Lane 1: protein marker. The size of protein marker and the VP6 protein is shown.

**Figure 7:**
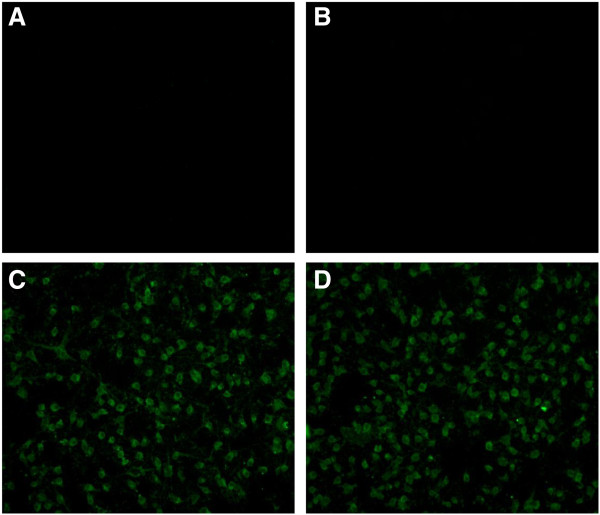
**Immunofluorescence assay of cell surface expression of VP6.** BHK cells were transfected with pVAX-VP6, then the cells were subjected to indirect immunofluorescence using anti-VP6 antiserum as primary antibody followed by incubation of FITC-conjugated secondary antibody (see M&M). A representative comparison is provided. **A**: vector-transfected cells with treatment of Triton X-100; **B**: vector-transfected cells without treatment of TritonX-100; **C**: VP6-transfected cells with treatment of Triton X-100; **D**: VP6-transfected cells without treatment of Triton X-100.

It is clea*r that E. coli* system has advantages such as low costs, high production, and manipulation convenience, etc. [15]. In our laboratory, we have expressed several heterologous proteins in this system [16-19]. In this study, we analyzed the bacterial expression of VP6 protein in *E.coli*. SDS-PAGE showed that fused VP6 protein was approx. 48 kDa as expected and accumulated in the bacteria as inclusion bodies (Figure 4). In this study, a rabbit anti-VP6 antibody was generated by conventional animal immunization. The optimization of the ELISA was determined. As shown in Figure 5, after the fixation of the concentration of PRV VP6 protein, the optimal antibody dilution was1:2^9^. In addition to reaction with VP6 protein, at such dilution, the anti-VP6 serum also had the best reactivity to PRV. The optimization of the ELISA may be useful reference to detect PRV from clinical samples in the future. The reactivity of the protein to anti-VP6 antibody was then analyzed by Western blot. Our result showed that the bacterially expressed VP6 can be recognized by the antiserum, confirming that the VP6 is both highly immunogenic and antigenic (Figure 6). The immunoreactivity of the anti-VP6 antibody to eukaryotically expressed VP6 was further analyzed by IFA. As shown in Figure 7, the antibody reacted with VP6-expressing cells; in contrast, no green signal was detected in mock-transfected cells, confirming the specific recognition of the anti-VP6 antibody. In this study, the anti-VP6 antibody was generated by inoculating rabbit with VP6 expressed from *E.coli*, however, this antibody reacted with VP6 protein transiently expressed on cells. These data further confirm that bacterially expressed VP6 can be used as a good immunogen and the antibody can be used as good diagnostic reagent for detection of PRV VP6 protein.

### Discrimination ELISA for detection of PRV

**Figure 8:**
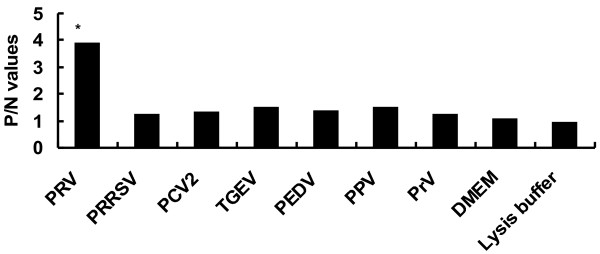
**Discrimination ELISA.** PRV, Porcine transmissible gastroenteritis virus (TGEV), Porcine epidemic diarrhea virus (PEDV), Porcine circovirus type II (PCV2), Porcine reproductive and respiratory syndrome virus (PRRSV), Porcine pseudorabies virus (PrV) and Porcine parvovirus (PPV), DMEM medium and virus lysis buffer were coated onto ELISA plates individually. The wells were incubated with the anti-PRV antibody followed by incubation of HRP-conjugated secondary antibody. The names of the pathogens are indicated in x axis; The OD_490_ value of tested samples (P)/the OD_490_ value of negative control, coating buffer (N)>2 is judged as positive. *compared with other group (p<0.01). The P/N values were the mean values from three independent assays.

Like PRV, TGEV and PEDV are capable of causing diarrhea symptoms in pigs. Other porcine viruses may cause co-infection with PRV, thus, discrimination between PRV and other viruses is important for clinical diagnosis purpose. Recently, we have confirmed that the antiserum to VP7 protein of PRV may also serve as a diagnostic agent for detection of PRV (unpublished data). Nevertheless, it has been pointed out that PRV VP6 protein is the most frequently target protein in diagnostic assays to detect virus particles. As ELISA is simple, convenient and sensitive immunological assays suitable for detection of pathogens [19-22], a discrimination ELISA for detection of PRV was established using the anti-VP6 antibody. Several control viruses were included in the ELISA to determine the specificity of the ELISA. The result showed that the anti-VP6 antibody had significant reactivity with PRV; in contrast, there was no positive P/N value among other controls (*p*<0.01) (Figure 8). Our results have demonstrated clearly that the VP6-antibody based ELISA works well in our laboratory, however, validation of the test using clinical samples are important so that the test can be applied in the field level for detection of PRVs. Thus, we will evaluate the utility of this VP6-antibody based ELISA in nature by including PRV clinical samples in the future.

## Methods

### Amplification of VP6 gene of PRV by PCR

PRV Chinese isolate named DN30209 was recently isolated in a pig farm in Heilongjiang Province, northeastern China. The virus was propagated in MA104 cells in presence of trypsin at a final concentration of 3 μg/mL (w/v). The viral total RNA was extracted with a commercial kit according to the manufacturer’s instructions (HaiGene, China). Sense primer (VP6-1): 5'- GGCTTTTAAACGAAGTCTTC and antisense primer (VP6-2): 5'- GGTCACATCCTCTCACTA were published primers [23] and used to amplify the VP6 gene for sequencing. The reverse transcription mixture included 5 μL of RNA (1 μg), 1 μL of primer VP6-2 (10 pmol), 4 μL of 5×RT-PCR buffer (TaKaRa, China),0.5 μL of RNase inhibitor, 1 μL of dNTP (10 mM/each), 1 μL of M-MLV reverse transcriptase (TaKaRa) and 7.5 μL of sterile water. The reaction was performed at 30°C for 10 min, 42°C for 60 min and 95°C for 5 min. Subsequent PCR mixture included the resulting 3 μL of cDNA (3 μg), 5 μL of 10×ExTaq buffer, 1 μL of each primer (10 pmol), 4 μL of dNTP (2.5 mM/each), 0.25 μL of ExTaqDNA (TaKaRa, China) and 35.75 μL of sterile water. The PCR was performed at 94°C for 5 min; 30 cycles of 94°C for 40 s, 52.8°C for 40 s, and 72°C for 1 min; a final extension at 72°C for 10 min. The PCR product was purified and subjected to DNA sequencing.

### RNA-polyacrylamide gel electrophoresis (PAGE)

RNA of the PRV was extracted by mixing the virus suspension in PBS or PBS (negative control) with an equal volume of an extraction buffer (0.02 M Tris–HCl pH7.4, 0.3 M NaCl, 0.01 M MgCl_2,_ 0.1% SDS, 5 μM EDTA, 4% sucrose and 0.04% bromophenol blue) followed by phenol–chloroform extraction at 12, 000 rpm and 4°C for 10 min. The resulting supernatant was subjected to RNA-PAGE to detect and analyze the genome of rotavirus. The RNA-PAGE was performed in 10% polyacrylamide slab gel using discontinuous buffer system without SDS as described by Laemmli [24] for 13 h at 10 mA. Viral RNA segments were visualized in silver staining.

### Sequence comparison and phylogenetic analysis

The obtained VP6 sequence was deposited in GenBank database and compared with reference PRV VP6 sequences using DNASTAR software. The name, GenBank accession number and virus isolation location of these genes are shown in Table 1. At the same time, a phylogenetic tree was constructed by neighbor-joining method base on these VP6 amino acid sequences using the DNASTAR program [25-27]. The bootstrap probabilities of each node were calculated using 1,000 replicates.

**Table 1:** Information of PRV VP6 genes used in this study

**Grouping**	**Virus name**	**Isolation place**
Procine rotavirus A	CRW-8	Australia
Procine rotavirus A	RU172	India
Procine rotavirus A	GD	China
Procine rotavirus A	4S	China
Procine rotavirus A	4F	China
Procine rotavirus A	JL94	China
Procine rotavirus A	DN30209	China
Rotavirus B stain	Bang373	Bangladesh
Human rotavirus	B219	Bangladesh
Procine rotavirus C	Cowden	USA
Human rotavirus C	WD534tc	USA
Human rotavirus C	Moduganari	Nigeria
Human rotavirus B	WH-1	China
Rotavirus C stain	Wu82	China
Procine rotavirus A	OSU	USA
Human rotavirus C	Preston	UK
Human rotavirus	TUCH	USA
Procine rotavirus A	Gottfried	USA

The grouping, name and origin of the PRV isolates used in this study are provided.

### Construction of recombinant plasmids bearing VP6 gene and its bacterial expression

To construct expression plasmids bearing the VP6 gene, forward primer: 5'- CCCCGGATCCATGCAAAATTACGGA(VP6F) and reverse primer (VP6R) 5'- GGGGAAGCTTGTTAGACTCGGTAATA were self designed and used to re-amplify the VP6 gene using the obtained PCR product above as template. Both primers contained BamH I and EcoR I sites, respectively. Subsequent PCR was performed as above using the full-length VP6 gene as template. The PCR profile included 95°C for 5 min; 30 cycles of 95°C for 30 s, 52.8°C for 30 s, and 72°C for 2 min as well as a final extension at 72°C for 10 min. The resulting PCR product was inserted into the prokaryotic expression vector pET30a and eukaryotic expression vector pVAX-1 to generate recombinant plasmids pET30a-VP6 and pVAX-VP6, respectively.

The pET30a-VP6 was transformed into *Escherichia coli* (*E. coli*) Rosetta. The transformed *E.coli* was cultured in Luria-Bertani (LB) medium supplemented with Kanamycin (50 mg/mL) with shaking at 37°C. When OD_600_ reached 0.6, isopropyl beta-D-thiogalactoside (IPTG) was added to the medium to a final concentration of 1 mM to induce protein expression. Control culture containing the same bacteria transformed with empty vector was used as control. The expressing protein designated as Pro-VP6 was subjected to gel-purification, sodium dodecyl sulfate-polyacrylamide gel electrophoresis (SDS-PAGE) and renaturation by dialysis according to our previously reported protocols [28].

### Generation of polyclonal antibody to VP6

Generation of polyclonal antiserum to Pro-VP6 was processed according to references with modifications [17-19,28]. A New Zealand rabbit was immunized with 2 mL of gel-purified Pro-VP6 (1 mg/mL) emulsified with equal amounts of Freund’s complete adjuvant via subcutaneous injection. After ten days, 2 mL of the same antigen mixed with Freund’s incomplete adjuvant were injected into the rabbit weekly at two intervals. Antiserum was collected from the peripheral blood of the rabbit.

The titration of the antiserum and its reaction with VP6 protein was determined using ELISA: Purified VP6 was coated onto ELISA plates at a final concentration of 5 μg/well at 4°C overnight. The next day, the wells were incubated with blocking buffer at 37°C for 2 h, after three washes with PBS-0.1% Tween20 (PBST). The wells were incubated with serially diluted polyclonal antibodies at 37°C for 1 h. Then the plates were incubated with goat anti-rabbit IgG conjugated with peroxidase (1:5000 dilution in PBS) at 37°C for 1 h, after three washes with PBST. OPD (*o*-Phenylenediamine dihydrochloride) substrate (100 μL/well) was added and incubated for 15 min followed by stopping reaction with 2 M H_2_SO_4_. The OD_490_ values were determined an ELISA reader. In addition, the reactivity of the anti-VP6 antibody to PRV was also tested. Briefly, the purified PRV (10^4^ PFU/mL) were coated onto ELISA plates (100 μL/well) and the dilution of antiserum to Pro-VP6 or the control serum was 1:2^4^, 1:2^5^, 1:2^6^, 1:2^7^, 1:2^8^, 1:2^9^, 1:2^10^, 1:2^11^ or 1:2^12^. The other experimental steps were performed as above.

### Western blot

Pro-VP6-containing bacterial protein and empty vector-transforming bacterial protein (negative control) were subjected to SDS-PAGE and transferred to a nitrocellulose (NC) membrane. Rabbit antiserum to Pro-VP6 was diluted 1:2,000 in PBS and was used to incubate the membrane at room temperature for 1.5 h followed by incubation with horseradish peroxidase (HRP)-conjugated goat anti-rabbit IgG (1:5000 dilution, Boster, China) for 1.5 h. The protein bands were visualized using OPD.

### Indirect immunofluorescence assay (IFA)

Baby hamster kidney (BHK) cells seeded onto 24-well plates were transfected with pVAX-VP6 using polyethylenimine (PEI) transfection agent (Sigma, China) according to the manufacturer’s instructions. Empty vector-transfected cells were used as control. The cells were fixed with 4% PFA (polyformaldehyde) in PBS at room temperature for 20 min and quenched with 0.1 M glycine in PBS. Then, the cells were washed three times with PBST and treated with or without 1% Triton X-100 for 10 min followed by incubation with anti-VP6 serum (1:200 dilution in PBS containing 0.1% BSA) for 1 h. After three washes with PBST, the cells were incubated with fluorescein isothiocyanate (FITC)-conjugated goat anti-rabbit IgG (1:200 in PBS containing 1% BSA) for 1 h at 37°C. After washing with PBST, the fluorescence signals were analyzed using fluorescence microscopy.

### Immunoreactivity of the anti-VP6 antibody to other control viruses

To evaluate the immunoreactivity of the anti-VP6 antibody, a discrimination ELISA was established. PRV, Porcine transmissible gastroenteritis virus (TGEV) strain HR/DN1, Porcine epidemic diarrhea virus (PEDV) strain HLJBY, Porcine circovirus type II (PCV2) strain PCV2-LJR, Porcine reproductive and respiratory syndrome virus (PRRSV) strain JilinTN1, Porcine pseudorabies virus (PrV) strain Kaplan and Porcine parvovirus (PPV) strain PPV2010 were treated with lysis buffer (0.1% SDS, 10 mmol/liter Tris–HCl [pH 7.4], and 1 mmol/liter EDTA) followed by dilution in carbonate–bicarbonate buffer (15 mM Na_2_CO_3_, 35 mM NaHCO_3_, pH 9.6) and the pathogens were coated into ELISA plates (8 μg/well) at 4°C overnight. Wells coated with DMEM or lysis buffer were used as control. The anti-PRV antibody was used as primary antibody in the above-mentioned ELISA to detect the pathogens. The OD_490_ value of tested samples (P)/the OD_490_ value of negative control, coating buffer (N)>2 was judged as positive. The experiment was performed in triplicate. Statistical analysis of the data was processed with SPSS 11.5 software; *p*<0.05 and *p*<0.01 were defined as statistically significant and statistically very significant, respectively.

## Competing interests

The authors have no competing interests.

## Authors' contributions

XR, JZ, QY, LC, XD, WZ, REB and YR carried out the molecular genetic studies, participated in the sequence alignment and drafted the manuscript. JZ, JZ, FD, GL and XR carried out the immunoassays. JZ, LC, GL, YR, SS and XR participated in the design of the study. XR conceived of the study and participated in its design and coordination and helped to draft the manuscript. All authors read and approved the final manuscript.
